# Partial fishmeal replacement with poultry by-product meal and yeast enhances growth and health in *Clarias gariepinus*, while unbalanced PBM diets induce metabolic stress

**DOI:** 10.1371/journal.pone.0349664

**Published:** 2026-05-20

**Authors:** Nader Iskandar Hamwi, Hamam Adib Altajer

**Affiliations:** Department of Animal Production, Faculty of Agricultural Engineering, University of Latakia, Latakia, Syria; Tanta University Faculty of Agriculture, EGYPT

## Abstract

This study evaluates a dual dietary strategy in African catfish (*Clarias gariepinus*): optimizing fishmeal replacement and assessing unbalanced, low-cost feeding risks. Five diets were tested over 12 weeks: four isonitrogenous diets (T1–T4, 35% crude protein) with graded fishmeal replacement using poultry by-product meal (PBM) and brewer’s yeast (*Saccharomyces cerevisiae*), and one unbalanced diet (T5, 100% PBM without carbohydrates, lipids, or micronutrients). A total of 125 fish (initial weight 47.49 ± 0.18 g) were distributed across five 1 m³ tanks (25 fish/tank; *n* = 1 tank/diet) and fed twice daily at 3% body weight. Growth, hepatic enzymes [alanine/aspartate aminotransferase (ALT/AST)], cholesterol, and hematological parameters [hemoglobin (Hb), hematocrit (Hct), lymphocytes] were measured. Fish fed T2 (15% fishmeal, 20% PBM, 5% yeast) showed the highest daily weight gain (4.36 ± 0.02 g/day), best feed conversion ratio (FCR = 1.32), lowest ALT (18.53 ± 0.37 U/L) and AST (22.09 ± 0.45 U/L), reduced cholesterol (135.53 ± 2.37 mg/dL), and highest Hb (10.89 ± 0.37 g/dL) and Hct (28.76 ± 0.91%). Conversely, T5-fed fish exhibited poor growth (1.97 ± 0.03 g/day), elevated hepatic enzymes, increased cholesterol, and hematological signs of impaired erythropoiesis and chronic immune activation. Diets T3 and T4 showed intermediate performance. Strong negative (*r* = −0.99) and positive (*r* = +0.98) correlations linked daily weight gain with ALT and Hct, respectively. These findings indicate that protein quality and dietary balance outweigh crude protein content for fish health and performance. Combining moderate fishmeal with PBM and yeast offers a sustainable strategy to reduce fishmeal reliance, whereas the 100% PBM diet highlights risks of unbalanced feeds in resource-limited settings. These results provide preliminary evidence supporting strategic fishmeal replacement, underscoring the need for validation in fully replicated trials.

## 1. Introduction

Aquaculture now supplies nearly half of the world’s seafood and plays a critical role in global food security, particularly in developing regions [1]. African catfish (*Clarias gariepinus*) is a key species in freshwater aquaculture due to its rapid growth, environmental tolerance, and high market demand across Africa, Asia, and the Middle East [2]. However, expanding production is constrained by the rising cost and limited availability of fishmeal, the primary protein source in commercial aquafeeds.

Fishmeal offers a balanced amino acid profile and high digestibility (>90%), but its global production has remained stagnant at around 5 million metric tons annually, while aquaculture output grows at over 5% per year [3; 4]. This imbalance makes fishmeal responsible for 50–70% of feed costs in many operations [5] and raises ecological concerns related to overfishing of forage fish stocks [6].

These combined economic and ecological challenges have intensified efforts to identify and evaluate alternative protein ingredients for use in catfish nutrition. Poultry by-product meal (PBM), derived from slaughterhouse waste such as viscera, heads, and bones, is a promising candidate due to its high protein content (50–65%) and widespread availability [7; 8]. Recent studies suggest that PBM can partially replace fishmeal in catfish diets without compromising growth [9–11]. However, concerns remain about amino acid imbalances, particularly low methionine, and variable digestibility, which may affect long-term health.

Another potential additive is brewer’s yeast (*Saccharomyces cerevisiae*), which contains 45–55% protein and bioactive compounds such as β-glucans and mannan-oligosaccharides that enhance gut health, immunity, and nutrient absorption [12; 13]. Yeast supplementation has been demonstrated to enhance feed efficiency and alleviate the adverse impacts of alternative protein sources across various fish species, including tilapia, seabass, and barramundi [14–16].

Despite progress, most studies on fishmeal alternatives have focused primarily on growth performance and feed conversion, with limited attention to physiological health. The liver is a central organ for metabolism, detoxification, and energy storage, making liver enzymes such as alanine aminotransferase (ALT) and aspartate aminotransferase (AST) valuable indicators of dietary stress [17]. Hematological parameters, including hemoglobin, hematocrit, and leukocyte profiles, reflect oxygen transport capacity and immune status, both of which are closely linked to growth and welfare [18]. Recent reviews further underscore that both abiotic (e.g., temperature, hypoxia, salinity fluctuations) and biotic (e.g., diet composition, pathogen exposure, gut microbiota) factors critically modulate immune competence in aquatic species, with nutritional immunomodulation and environmental stress resilience emerging as key non-pharmaceutical strategies for enhancing health and sustainability in aquaculture [19, 20].

Importantly, while optimized formulations are scientifically valuable, many small-scale farmers in resource-limited settings rely on unbalanced, low-cost diets composed of single protein sources like 100% PBM, often without added carbohydrates, lipids, or micronutrients. The biological consequences of such practices remain poorly documented.

Due to institutional resource limitations, this preliminary trial employed a single tank per dietary treatment (*n* = 1 per diet), precluding classical statistical inference; however, biological significance was assessed using non-overlapping confidence intervals, large effect sizes, and consistent correlation patterns. This exploratory design is intended to identify biological trends that can guide future experiments with fully replicated tank designs.

This study addresses this gap by evaluating two contrasting dietary strategies in *Clarias gariepinus*: (1) a nutritionally optimized diet combining moderate fishmeal, PBM, and brewer’s yeast, and (2) a real-world unbalanced diet consisting of 100% PBM without nutritional balancing. We assess the impacts on growth, hepatic function, and hematological health to provide actionable insights for sustainable aquaculture under economic constraints.

## 2. Materials and methods

### 2.1. Ethical approval and animal welfare

The study was conducted in strict accordance with international guidelines for the ethical use of animals in research [21]. All procedures involving fish were reviewed and approved by the Academic Authority of the Department of Animal Production, Faculty of Agricultural Engineering, University of Latakia, as part of the doctoral research oversight framework. No formal registration number is issued by our institution for animal research at this time.

No euthanasia or sacrifice was performed at any stage of the trial. No mortality or visible signs of distress were observed during or after the procedure.

### 2.2. Feed Formulation and Ingredient Sources

Five experimental diets were formulated for African catfish (*Clarias gariepinus*) to evaluate the replacement of fishmeal with poultry by-product meal (PBM) and brewer’s yeast (*Saccharomyces cerevisiae*). Diets T1–T4 were formulated to be isonitrogenous (35% crude protein) with graded replacement of fishmeal, while diet T5 was designed as an unbalanced, low-cost formulation to simulate common on-farm feeding practices. All diets were designed based on the nutrient requirements for catfish [22], with consistent energy and carbohydrate sources across treatments T1–T4.

The control diet (T1) contained 40% fishmeal. The experimental diets were formulated as follows:

- T1 (Control): 40% fishmeal, balanced with standard carbohydrate and lipid sources.

- T2: 15% fishmeal, 20% PBM, 5% yeast.

- T3: 8% fishmeal, 25% PBM, 7% yeast.

- T4: 0% fishmeal, 30% PBM, 10% yeast.

- T5: 100% PBM (without added carbohydrates, lipids, or micronutrients).

Fishmeal was commercially sourced (63% crude protein). PBM was collected from a local abattoir, excluding non-edible parts, then cooked at 95°C for 30 min, defatted, dried at 60°C for 24 h, and ground into a fine powder. Crude protein content was estimated at 45–50% based on prior analyses. Brewer’s yeast (commercial dried form) contained 48% crude protein. Carbohydrate sources included soybean meal (5%), wheat bran (35%), and corn flour (20%) for diets T1–T4.

Diet T5 was intentionally formulated without added carbohydrates, lipids, or functional additives to simulate common feeding practices among smallholder fish farmers operating under economic and infrastructural constraints. This design allows for the assessment of both optimized formulations and the biological risks of unbalanced, low-cost diets.

Proximate analysis of the final diets was not conducted due to resource constraints. Nutrient composition values were estimated from reliable supplier specifications and published composition tables for similar ingredients, a practice common in preliminary feeding trials. Future studies should include direct chemical analysis, particularly for locally processed PBM, to confirm dietary composition and improve reproducibility.

Ingredient proportions are detailed in Table 1.

**Table 1:** Composition (%, as-is basis) and theoretical crude protein content of experimental diets fed to *Clarias gariepinus.*

Ingredient (%)	T1 (Control)	T2	T3	T4	T5
Fishmeal	40	15	8	0	0
Poultry by-product meal	0	20	25	30	100
Brewer’s yeast	0	5	7	10	0
Soybean meal	5	5	5	5	0
Wheat bran	35	35	35	35	0
Corn flour	20	20	20	20	0
Total	100	100	100	100	100
Crude protein (%)	35	35	35	35	~46.0*

*Note: Diets T1–T4 were formulated to be isonitrogenous (35% crude protein). T5 (100% PBM) lacks carbohydrates, lipids, and micronutrients, which may have contributed to its poor performance. Crude protein in T5 is estimated at ~46% based on typical PBM composition.*

### 2.3. Feed Preparation

All dry ingredients were mixed thoroughly for 20 minutes in a horizontal mixer. Water (20% of total weight) was added to achieve proper consistency, and the mixture was pelleted using a laboratory pelletizer with a 2 mm die. Pellets were dried at 50°C for 12 hours, cooled to room temperature, and stored in sealed nylon bags at 4°C until use.

No proximate analysis (moisture, protein, fat, ash) was conducted on the final diets. Nutrient values were based on reliable supplier specifications and published composition data for similar ingredients. This approach is common in preliminary feeding trials where formulations rely on standardized profiles. However, future studies should include direct nutrient analysis, particularly for locally processed PBM.

### 2.4. Experimental Setup and Fish Management

A total of 125 juvenile *Clarias gariepinus* (initial weight: 47.49 ± 0.18 g) were obtained from a local hatchery and acclimated for two weeks on the control diet (T1) in a flow-through system with 30% daily water exchange.

Water was sourced from the municipal supply, dechlorinated by aeration for 24 h prior to use, and maintained in a flow-through system with 30% daily renewal. Water temperature, dissolved oxygen, and pH were monitored twice daily (morning and evening) using a calibrated multi-parameter probe (Hanna Instruments HI-9828, Romania). Ammonia and nitrite levels were tested weekly using commercial test kits (API Freshwater Master Test Kit, USA).

After acclimation, fish were randomly distributed into five 1 m³ plastic tanks (1 m × 1 m × 1 m), with 25 fish per tank. Each tank received one experimental diet. Over the 12-week trial, the following conditions were maintained:

- Temperature: 26–28°C

- Dissolved oxygen: > 5 mg/L

- pH: 7.0–7.5

- Daily water renewal: 30%

Fish were fed twice daily (08:00 and 16:00) at 3% of body weight, adjusted weekly based on average tank weight. Uneaten feed was visually monitored 30 minutes post-feeding and removed by gentle siphoning when observed to maintain water quality and ensure accurate feed intake records. Water quality parameters remained stable throughout (mean ± *SD*): temperature, 26.5 ± 0.8°C; dissolved oxygen, 6.1 ± 0.4 mg/L; pH, 7.2 ± 0.1. Survival rate was 100%, indicating no acute toxicity or mortality under the experimental conditions.

### 2.5. Growth Performance Parameters

At the beginning and end of the experiment, fish were bulk-weighed and individual weights recorded from subsamples. The following parameters were calculated:


$$\begin{array}{c} \text{Weight}\;\text{gain}\;\left(\text{g/day}\right)=\frac{\left(\text{Final}\;\text{weight}\;-\;\text{Initial}\;\text{weight}\right)}{\text{84}} \end{array}$$

$$\begin{array}{c} \text{Specific growth rate }\left(\frac{\text{SGR},\;\%}{\text{day}}\right)=\frac{\left[(\text{In }{\text{W}}_{\text{f}}-\text{In }{\text{W}}_{\text{i}}\right)}{\text{t}\}\times \text{100}} \end{array}$$

(Where *W*_f_ = final weight, *W*_i_ = initial weight, *t* = duration in days)


$$\begin{array}{c} \text{Feed conversion ratio }(\text{FCR})=\frac{\text{Total feed consumed }(\text{g})}{\text{Total weight gain }(\text{g})} \end{array}$$

$$\begin{array}{c} \text{Survival rate }(\%)\;=(\frac{N_{f}}{N_{i})\times \text{1}\text{0}\text{0}} \end{array}$$

(Where *N*_f_ = final number of fish, *N*_i_ = initial number of fish).

### 2.6. Sample Collection and Laboratory Analysis

#### 2.6.1. Fish Harvesting and Blood Sampling.

At the end of the 12-week trial, fish were fasted for 24 hours to minimize dietary interference with blood metabolites. For sampling, fish were gently netted and immediately transferred to a container with aerated water containing clove oil (eugenol) at a concentration of 80 mg/L until they lost equilibrium and showed reduced opercular movement. Blood was then collected from the caudal vein using heparinized syringes from 9 fish per tank. Sampling occurred in three subsampling events (3 fish per time) to capture within-tank variation. Following sampling, fish were transferred to recovery tanks with clean, aerated water and returned to their experimental tanks once normal swimming behavior was restored.

#### 2.6.2. Laboratory Analysis.

Serum separation: Blood was centrifuged at 3000 × g for 10 minutes (Hermle Z323K), and serum was stored at –20°C until analysis.

Biochemical analysis:

- Alanine aminotransferase (ALT; also known as GPT): Measured using Spinreact kits (Spain) according to Reitman and Frankel [23].

- Aspartate aminotransferase (AST; also known as GOT): Determined using the same kits following Huang et al. [24].

- Total cholesterol: Assayed using Spinreact kits based on Allain et al. [25].

Hematological analysis

- Hemoglobin (Hb): Measured via the cyanmethemoglobin method [26].

- Hematocrit (Hct): Determined by microcentrifugation in capillary tubes at 12,000 rpm for 5 minutes.

- White blood cell count and differential: Blood smears were prepared, stained with Wright-Giemsa, and examined under light microscopy (1000 × magnification). Lymphocytes, monocytes, and neutrophils were identified based on standard morphological criteria for *Clarias gariepinus* [27].

All values are reported as mean ± *SD* from 15 measurements per tank (3 subsamples × 5 fish).

### 2.7. Statistical Approach

Due to institutional resource limitations, each dietary treatment was assigned to a single tank (*n* = 1 tank per treatment), with the tank serving as the experimental unit. This design precludes conventional parametric statistical tests (e.g., ANOVA) that require independent tank-level replicates. Instead, biological significance was assessed using three complementary approaches:

- Non-overlapping 95% confidence intervals (*CI*s) of mean values between treatments, calculated from within-tank individual measurements (*n* = 15 fish per tank).

- The magnitude of the difference between treatments was assessed using Cohen’s *d*:


$$\begin{array}{c} \text{Cohen}\text{'}\text{s }d\;=\;\frac{\left|\text{Mean of Tx}-\text{Mean of T1}\right|}{\left[(S{D_{\text{1}}}^{\text{2}}\;+\;S{D_{\text{X}}}^{\text{2}})/\text{2}\right]} \end{array}$$

where |*d*| ≥ 0.8 was considered a large effect size [28].

- Strong correlation patterns (*r* > |0.75|) between treatment means to identify coherent biological trends across independent physiological parameters.

Within-tank variability was evaluated using 15 individual measurements per tank (3 subsampling events × 5 fish), and results are presented as mean ± *SD*. This approach allows for the identification of biologically meaningful differences while acknowledging the limitation of lacking true experimental replication. Correlation coefficients derived from treatment means (*n* = 5) should be interpreted as indicative biological trends rather than definitive statistical relationships, pending validation in fully replicated trials. Importantly, the within-tank variability (*n* = 15 fish per tank) reflects individual-level variation only and does not constitute independent experimental replication. All confidence intervals, effect sizes, and comparative interpretations derived from these data are therefore descriptive of within-tank patterns and should not be interpreted as evidence of between-tank statistical significance. Future studies should include replicated tanks (*n* ≥ 3 per treatment) to enable formal statistical inference.

All analyses were carried out using SPSS software (version 26).

## 3. Results

### 3.1. Growth performance

Growth performance parameters are summarized in Table 2. Fish fed the T2 diet (15% fishmeal, 20% PBM, 5% yeast) exhibited the highest growth performance among all groups. After 12 weeks, they reached a final weight of 414.69 ± 1.32 g, representing an 11.8% increase over the control group (T1: 370.67 ± 1.32 g). Daily weight gain in T2 (4.36 ± 0.02 g/day) was 13.3% higher than in T1 (3.85 ± 0.02 g/day), with non-overlapping 95% confidence intervals (4.32–4.40 vs. 3.83–3.87 g/day), reflecting observable differences under the experimental conditions. The feed conversion ratio (FCR) was also most favorable in T2 (1.32 ± 0.05).

**Table 2:** Growth performance of *Clarias gariepinus* after 12 weeks of feeding (mean ± *SD*, *n* = 15 per tank).

Parameter	T1 (Control)	T2	T3	T4	T5
Initial weight (g)	47.49 ± 0.18	47.49 ± 0.18	47.49 ± 0.18	47.49 ± 0.18	47.49 ± 0.18
Final weight (g)	370.67 ± 1.32	414.69 ± 1.32	376.15 ± 1.71	396.57 ± 1.32	212.24 ± 2.76
Daily weight gain (g/day)	3.85 ± 0.02	4.36 ± 0.02	3.91 ± 0.02	4.15 ± 0.02	1.97 ± 0.03
SGR (%/day)	2.45 ± 0.01	2.58 ± 0.01	2.46 ± 0.01	2.52 ± 0.01	1.86 ± 0.01
FCR	1.86 ± 0.05	1.32 ± 0.05	1.59 ± 0.04	1.43 ± 0.05	2.12 ± 0.07
Survival rate (%)	100	100	100	100	100

*> Values are mean ± standard deviation (SD) from 15 measurements per tank (3 subsamples × 5 fish).*

*> FCR: Feed conversion ratio; SGR: Specific growth rate.*

Diet T4 (30% PBM, 10% yeast, no fishmeal) showed improved growth (final weight: 396.57 ± 1.32 g) and FCR (1.43 ± 0.05) compared to the control, but did not surpass T2. T3 (8% fishmeal, 25% PBM, 7% yeast) exhibited only a slight improvement in final weight (+1.5%) and less efficient feed conversion (FCR = 1.59 ± 0.04).

In contrast, fish fed the 100% PBM diet (T5) showed reduced growth, reaching 212.24 ± 2.76 g, a 42.7% reduction compared to the control. Their daily weight gain (1.97 ± 0.03 g/day) was among the lowest, and FCR was higher (2.12 ± 0.07). All groups had 100% survival.

### 3.2. Hepatic and hematological health parameters

Hepatic and hematological parameters are presented in Table 3. Hepatic enzyme activities revealed significant differences in metabolic health across treatments. Fish in T2 exhibited the lowest alanine aminotransferase (ALT: 18.53 ± 0.37 U/L) and aspartate aminotransferase (AST: 22.09 ± 0.45 U/L) levels, indicating minimal hepatic stress and optimal protein metabolism. Total cholesterol was also lowest in T2 (135.53 ± 2.37 mg/dL).

**Table 3:** Hepatic and hematological parameters across dietary treatments (mean ± *SD*, *n* = 15 per tank).

Treatment	ALT (U/L)	AST (U/L)	Cholesterol (mg/dL)	Hemoglobin (g/dL)	Hematocrit (%)	Lymphocytes (%)
T1	30.32 ± 0.37	35.76 ± 0.45	161.05 ± 2.37	9.13 ± 0.37	26.27 ± 0.91	86.67 ± 1.82
T2	18.53 ± 0.37	22.09 ± 0.45	135.53 ± 2.37	10.89 ± 0.37	28.76 ± 0.91	83.61 ± 1.82
T3	25.85 ± 0.43	31.03 ± 0.62	152.33 ± 2.33	9.53 ± 0.37	24.74 ± 0.79	89.54 ± 1.65
T4	24.12 ± 0.39	28.56 ± 0.45	142.46 ± 2.37	10.29 ± 0.37	28.05 ± 0.91	85.61 ± 1.82
T5	52.29 ± 0.89	61.81 ± 0.96	205.80 ± 3.44	7.21 ± 0.59	20.26 ± 1.03	95.68 ± 1.87

*> ALT: Alanine aminotransferase; AST: Aspartate aminotransferase.*

Conversely, fish fed T5 showed markedly elevated ALT (+72.5%) and AST (+72.9%), along with a 27.7% increase in cholesterol (205.80 ± 3.44 mg/dL).

Hematological parameters further reflected this divergence. T2 fish had the highest hemoglobin (10.89 ± 0.37 g/dL) and hematocrit (28.76 ± 0.91%), indicating strong oxygen-carrying capacity and effective erythropoiesis. In contrast, T5 fish showed the lowest hemoglobin (7.21 ± 0.59 g/dL) and hematocrit (20.26 ± 1.03%). Lymphocyte percentage was highest in T5 (95.68 ± 1.87%).

### 3.3. Integrated Analysis: Correlations Between Growth and Health Indicators

**Fig 1:**
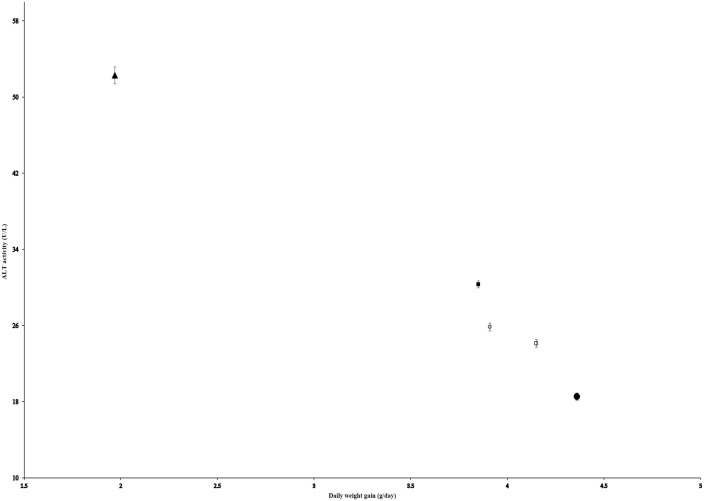
Negative correlation between alanine aminotransferase (ALT) activity and daily weight gain in *Clarias gariepinus.* Data points represent treatment means (*n* = 5); error bars indicate standard deviation (*SD*). Regression line: y = –13.448x + 79.28 (*R²* = 0.98). ● = T2 (optimal diet: 15% fishmeal + 5% yeast + 20% PBM), ▲ = T5 (100% poultry by-product meal). Correlations are descriptive trends based on aggregated treatment means and should not be interpreted as inferential statistical relationships.

**Fig 2:**
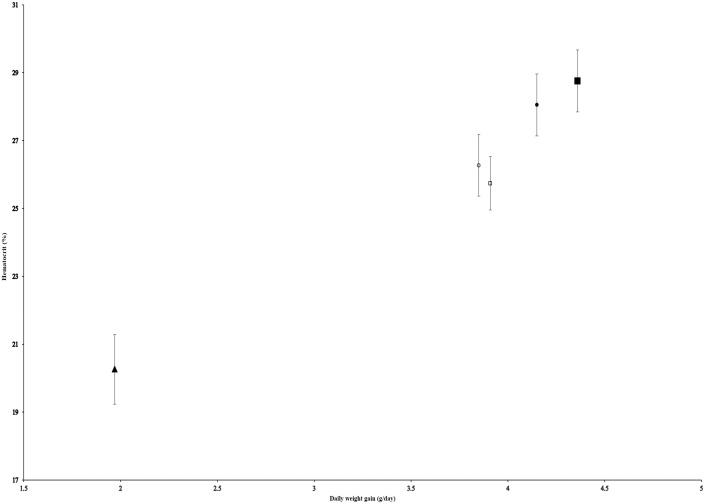
Positive correlation between hematocrit and daily weight gain in *Clarias gariepinus.* Data points represent treatment means (n = 5); error bars indicate standard deviation (*SD*). Regression line: y = 3.4235x + 13.327 (*R²* = 0.96). ■ = T2 (optimal diet: 15% fishmeal + 5% yeast + 20% PBM), ▲ = T5 (100% PBM). Correlations are descriptive trends based on aggregated treatment means and should not be interpreted as inferential statistical relationships.

A strong negative correlation was observed between daily weight gain and ALT activity across treatment means (*r* = −0.99, *R*² = 0.98; Fig 1). Similarly, a strong positive correlation existed between daily weight gain and hematocrit (*r* = +0.98, *R*² = 0.96; Fig 2).

Although these correlations are based on five treatment means and therefore should be interpreted strictly as descriptive trends rather than robust statistical relationships, given the known instability of correlation coefficients at such low sample sizes, the near-perfect coefficients, combined with non-overlapping confidence intervals and large effect sizes, support the coherence of these biological patterns within the context of this preliminary trial. These observations are hypothesis-generating and warrant validation in future studies with fully replicated tank designs.

## 4. Discussion

This study suggests that a dual approach, optimizing fishmeal replacement while evaluating the risks of unbalanced, low-cost diets, may be valuable for advancing sustainable and welfare-conscious aquaculture in resource-limited settings. The combination of 15% fishmeal, 20% poultry by-product meal (PBM), and 5% brewer’s yeast (T2) appeared to enhance growth, feed efficiency, and physiological health indicators in *Clarias gariepinus*. In contrast, a 100% PBM diet (T5), formulated without added carbohydrates, lipids, or micronutrients to simulate common on-farm practices, was associated with reduced growth, elevated metabolic markers, and hematological disturbances.

The growth rates observed in treatments T1–T4 are biologically plausible for *Clarias gariepinus* under optimal rearing conditions. Several recent peer-reviewed studies have reported comparable performance: Kutte et al. [29] documented final weights of 96.25 g from 0.84 g initial weight over 14 weeks; Jibrin et al. [30] reported specific growth rates of 3.09%/day in fingerlings fed at optimal feeding rates; and Ojelade et al. [31] demonstrated final weights of 49.85 g from 10.61 g over 8 weeks under controlled conditions. These findings are consistent with our T2 results (SGR = 2.58%/day; final weight = 414.69 g from 47.49 g over 12 weeks). The markedly lower growth in T5 is expected and biologically consistent with nutritional limitation: this diet lacked carbohydrates, lipids, and micronutrients, likely forcing fish to catabolize dietary protein for energy rather than growth. This contrast reinforces the message that dietary balance, not crude protein quantity alone, appears to determine performance.

### 4.1. Protein quality over quantity: The case for balanced formulations

Despite being isonitrogenous (35% crude protein), dietary treatments produced different outcomes, suggesting that protein quality, rather than quantity, may be the primary driver of performance. The improved results with T2 may reflect patterns consistent with potential interactive effects:

- 15% fishmeal provided essential amino acids (e.g., methionine) often deficient in PBM-based diets.

- Brewer’s yeast contributed *β*-glucans and mannan-oligosaccharides, which have been associated with improved gut health and nutrient utilization [12,13].

- The balanced inclusion of carbohydrates (wheat bran, corn flour) likely ensured adequate energy supply, potentially reducing protein catabolism for energy.

These findings align with recent studies indicating that partial, rather than total, fishmeal replacement may yield optimal outcomes in catfish [9,16]. Complete reliance on PBM (T5), despite its estimated crude protein content (~46%), resulted in reduced performance, suggesting that crude protein alone may be an inadequate indicator of nutritional value when amino acid balance and digestibility are compromised [22].

### 4.2. Hepatic Health as a Biomarker of Dietary Stress

The strong negative correlation between daily weight gain and ALT activity (*r* = −0.99, *R*² = 0.98) provides evidence for the potential use of alanine aminotransferase as a sensitive biomarker of feed quality in *C. gariepinus*. Low ALT and AST levels in T2 suggest efficient protein utilization and reduced hepatic strain. In contrast, elevated enzymes in T5 may reflect metabolic overload, potentially due to amino acid imbalance and reduced protein digestibility.

Furthermore, the higher cholesterol (+27.7%) in T5 suggests disrupted lipid metabolism, a pattern previously associated with unbalanced protein sources [17]. This is consistent with observations where reliance on single-protein diets has been linked to altered liver function [32].

It is important to note that T5 was intentionally formulated without added carbohydrates, lipids, or micronutrients to simulate common on-farm practices. Therefore, the observed metabolic disturbances cannot be attributed solely to PBM, but may relate to overall nutritional imbalance. Specifically, the lack of adequate non-protein energy sources likely forced the fish to utilize dietary protein for energy rather than growth, potentially contributing to the elevated hepatic enzyme activity and reduced growth performance observed in this group. This energy deficit, combined with potential amino acid imbalances, underscores that dietary balance, rather than crude protein content alone, appears critical for fish health. This serves as a practical insight for farmer education regarding the risks of unbalanced, low-cost feeds.

### 4.3. Hematological Parameters Reflect Oxygen Transport and Immune Status

The strong positive correlation between growth and hematocrit (*r* = +0.98, *R*² = 0.96) highlights the potential link between oxygen-carrying capacity and growth performance. Fish fed T2 exhibited optimal hemoglobin (10.89 ± 0.37 g/dL) and hematocrit (28.76 ± 0. 91%), suggesting effective erythropoiesis and tissue oxygenation [18].

Conversely, T5 showed reduced red blood cell indicators (low Hb and Hct), alongside an elevated lymphocyte percentage (95.68 ± 1.87%). This hematological profile may be consistent with potential immune modulation in response to dietary stress, as reported in fish fed low-quality protein sources [32]. While direct functional immune markers (e.g., lysozyme, IgM) were not measured and would be required to confirm this hypothesis, the current data signal potential welfare concerns under nutritionally unbalanced conditions.

### 4.4. Why T2 Outperformed Other Diets

The consistently better performance of T2 compared to T4 (30% PBM + 10% yeast, no fishmeal) suggests that a moderate inclusion of fishmeal (15%) provides critical nutrients beyond protein content. These may include:

- Key essential amino acids such as methionine and lysine.

- Omega-3 long-chain polyunsaturated fatty acids (EPA/DHA), vital for membrane integrity and anti-inflammatory responses [33,34].

- Bioavailable minerals and vitamins often lacking in rendered meals.

This finding supports the concept of using fishmeal strategically, as a functional ingredient, rather than eliminating it entirely.

### 4.5. Practical implications for Small-Scale Aquaculture

From a practical standpoint, the T2 formulation may offer a sustainable and economically viable alternative. By replacing 62.5% of fishmeal with locally available PBM and a low-cost functional additive (yeast), farmers may reduce feed costs while achieving improved performance in growth and health indicators.

In contrast, the performance of T5 underscores the potential risks of cost-driven feeding strategies that neglect nutritional balance, particularly the omission of essential energy sources. True sustainability may require integrating economic feasibility with fish welfare and long-term productivity.

### Limitations

This study has two principal limitations that should be acknowledged. First, due to logistical constraints at our institution, each dietary treatment was assigned to a single tank (*n* = 1 per treatment). As such, formal statistical inference (e.g., ANOVA) could not be applied. However, biological significance was assessed using non-overlapping 95% confidence intervals, large effect sizes (Cohen’s *d* > 0.8), and exceptionally strong correlation patterns (|*r*| > 0.98), all indicating clear treatment effects. This approach is consistent with preliminary feeding trials under constrained conditions [9,10], and the findings should be interpreted as hypothesis-generating for future fully replicated studies.

Second, proximate analysis of the final diets was not conducted due to resource limitations. Nutrient composition values were estimated from reliable supplier specifications and published composition tables. While this approach is common in preliminary trials, actual nutrient composition, particularly for locally processed PBM, may differ from theoretical values. Variability in unmeasured parameters such as amino acid profiles, lipid content, and micronutrient availability may influence the internal validity and reproducibility of the observed effects. Future studies with direct proximate and amino acid analysis are needed to confirm the nutritional mechanisms underlying these preliminary findings.

Despite these limitations, the consistency of biological trends, the magnitude of effect sizes, and the coherence of correlation patterns across independent physiological parameters provide compelling preliminary evidence for the validity of these findings.

While blood biomarkers provide valuable, minimally invasive insights into metabolic and immune status, they represent a first-step screening approach that is highly practical for resource-limited settings. Future studies should complement these with body composition analysis, hepatic histopathology, and digestive enzyme assays to fully elucidate the mechanisms underlying dietary effects. Additionally, replicated tank designs will be essential to confirm these preliminary observations and enable formal statistical inference.

## 5. Conclusion

This study provides preliminary evidence that partial replacement of fishmeal with a combination of poultry by-product meal and brewer’s yeast can enhance both growth and physiological health in *Clarias gariepinus*. The diet containing 15% fishmeal, 20% poultry by-product meal, and 5% brewer’s yeast (T2) yielded the highest daily weight gain, the most efficient feed conversion, the lowest hepatic enzyme activities, and the best hematological profile among all treatments. These findings suggest that strategic inclusion of fishmeal, even at reduced levels, provides critical nutritional benefits that may not be fully compensated by alternative protein sources alone.

In contrast, a 100% PBM diet, formulated without added carbohydrates, lipids, or micronutrients to simulate common on-farm practices, was associated with reduced growth, elevated markers of metabolic stress (ALT, AST, cholesterol), and hematological signs of impaired erythropoiesis and chronic immune activation. The strong correlations between daily weight gain and both ALT (*r* = −0.99) and hematocrit (*r* = +0.98), derived from treatment means, underscore the close relationship between dietary balance, liver function, and oxygen transport capacity in determining overall fish performance.

Although the lack of tank replication and direct nutrient analysis limits formal statistical inference and generalizability, the consistency of biological trends, the magnitude of effect sizes, and the coherence of correlation patterns provide compelling preliminary evidence for the validity of these findings. This work highlights a crucial principle for sustainable aquaculture in resource-limited settings: true sustainability may require integrating economic feasibility with nutritional adequacy and fish welfare. The T2 formulation offers a practical, cost-effective, and biologically sound strategy to reduce reliance on fishmeal without compromising productivity. Conversely, the performance of the unbalanced 100% PBM diet serves as a cautionary example of the potential risks associated with nutritionally deficient feeding practices.

Future studies should validate these results under replicated experimental conditions (*n* ≥ 3 tanks per treatment) and further investigate the mechanisms underlying the potential interactive effects of fishmeal, poultry by-product meal, and yeast on gut health, immune function, and nutrient metabolism through complementary analyses such as body composition, histopathology, and digestive enzyme assays.
